# Whole-genome sequencing of *Escherichia coli* DH5α using MinION nanopore technology

**DOI:** 10.1128/mra.01076-25

**Published:** 2026-03-12

**Authors:** Megha S. Kumar, P.V. Suneesh, Manoj Bhat Krishna, K.P. Soman, T.G. Satheesh Babu

**Affiliations:** 1 Department of Chemistry, Amrita School of Physical Sciences, Amrita Vishwa Vidyapeetham, Coimbatore, India; 2 Amrita Biosensor Research Lab, Amrita School of Engineering, Amrita Vishwa Vidyapeetham, Coimbatore, India; 3 Amrita School of Artificial Intelligence, Amrita Vishwa Vidyapeetham, Coimbatore, India; 4 Amrita Biomedical Engineering Centre, Amrita School of Engineering, Amrita Vishwa Vidyapeetham, Coimbatore, India; Loyola University Chicago, Chicago, Illinois, USA

**Keywords:** whole-genome sequencing, *E. coli*, *de novo* assembly, MinION

## Abstract

We present the whole-genome sequence of *Escherichia coli* DH5α, a laboratory strain integral to molecular biology applications. We assembled a high-quality genome using Qiagen’s DNeasy Blood and Tissue Kit and the Oxford Nanopore MinION platform (~4.58 Mb, 50.8% guanine-cytosine [GC] content), offering insights into genetic structure and functional characteristics.

## ANNOUNCEMENT

*Escherichia coli* is a gram-negative bacteria commonly associated with warm-blooded animals and a core member of the *Enterobacteriaceae* family (1). The species exhibits considerable genomic and phenotypic diversities shaped by adaptation to distinct ecological niches, resulting in strain-specific metabolic capabilities and stress responses (2, 3). This makes *E. coli* an excellent model organism for investigating bacterial physiology, genetics, and evolution.

*E. coli* DH5α is a widely used laboratory strain derived from the K-12 lineage and engineered for high-efficiency cloning applications (4). Its key genetic features include the lacZΔM15 mutation enabling blue-white screening, the recA1 and endA1 mutations that improve insert stability and plasmid DNA quality, and the hsdR17 allele, which permits efficient transformation of methylated DNA (4). A closed genome sequence has previously been reported for NEB 5-alpha, a commercial *fhuA2* derivative of DH5α that serves as a finished reference for this lineage (5). However, NEB 5-alpha represents a proprietary laboratory derivative rather than a culture-collection DH5α isolate. To provide an independent long-read reference genome for a widely distributed DH5α strain, this study sequenced and assembled *E. coli* DH5α obtained from the Microbial Type Culture Collection (MTCC 1652), Chandigarh, India.

Cells were cultured in Luria-Bertani broth at 37°C with shaking at 200 rpm for 10 h before DNA extraction. Genomic DNA was isolated using the DNeasy Blood and Tissue Kit (Qiagen) following the protocol for gram-negative bacteria. No deliberate DNA fragmentation step was performed; high-molecular-weight DNA was preserved throughout extraction and library preparation, with only minimal mechanical stress introduced during routine pipetting and clean-up steps. Library preparation was performed with the Ligation Sequencing Kit (SQK-LSK109, Oxford Nanopore Technologies [ONT]), and sequencing was conducted on the MinION platform with a FLO-MIN106 flow cell (R9.4.1). AMPure XP bead clean-up was performed using short fragment buffer to retain fragments across the full size distribution without additional size selection. Read acquisition and quality were monitored using MinKNOW, and high-accuracy basecalling with adapter trimming was performed using Guppy v3.4.4 with the dna_r9.4.1_450bps_hac.cfg configuration.

Sequencing produced 81,605 reads with a read length N50 of 10,380 bp, a mean read length of 5,974 bp, a mean Phred quality score of 11.1, and an estimated genome coverage of 93×, as assessed using NanoPlot (6). *De novo* assembly was performed with Unicycler v0.5.0 (7) in long-read-only mode, followed by three rounds of polishing with Racon. The circular chromosome was automatically rotated by Unicycler during assembly to begin at the dnaA gene. Additional consensus polishing was performed using Medaka v1.12.1 (8) with the r941_min_high_g306 model. The genome annotation was performed with National Center for Biotechnology Information (NCBI) Prokaryotic Genome Annotation Pipeline (PGAP) v6.10 (9). All the bioinformatics tools used default parameters, unless otherwise stated.

The final assembly comprises a single circular chromosome of 4,583,112 bp with a guanine-cytosine (GC) content of 50.84% and no plasmids. PGAP identified 4,542 genes, including 4,421 protein-coding sequences and 121 RNA genes. Two CRISPR arrays were detected using CRISPRCasFinder v4.3.2 (10), with consensus repeat sequences GTGTTCCCCGCGCCAGCGGGGATAAACC and GAGTTCCCCGCGCCAGCGGGGATAAACC, which are consistent with conserved type I-E CRISPR repeats in *E. coli* (11).

## Data Availability

The raw reads are available under accession number SRR31302084, and the assembled genome is available under accession number CP199401.1 in NCBI under BioProject accession number PRJNA1184340.
